# The Social Determinants of Health and the Decline in U.S. Life Expectancy: Implications for Appalachia

**DOI:** 10.13023/jah.0101.02

**Published:** 2019-04-30

**Authors:** Steven H. Woolf, Heidi Schoomaker, Latoya Hill, Christine M. Orndahl

**Affiliations:** Virginia Commonwealth University, steven.woolf@vcuhealth.org; VCU Health, Heidi.Schoomaker@vcuhealth.org; VCU Health, Latoya.Hill@vcuhealth.org; Virginia Commonwealth University, orndahlc@mymail.vcu.edu

**Keywords:** Social Determinants of Health, life expectancy, Appalachia

## Abstract

For the past century, life expectancy in industrialized countries has increased, and the U.S. has shared in that progress. However, beginning in the 1980s, advances in U.S. life expectancy began to lose pace with peer countries. By 1998, U.S. life expectancy had fallen below the average for Organisation for Economic Cooperation and Development nations. U.S. life expectancy peaked in 2014 and has been decreasing for three consecutive years, a trend not been seen since the influenza pandemic a century ago. Put simply, U.S. health is in decline.

For the past century, life expectancy in industrialized countries has increased, and the U.S. has shared in that progress. However, beginning in the 1980s, advances in U.S. life expectancy began to lose pace with peer countries.^1^ By 1998, U.S. life expectancy had fallen below the average for Organisation for Economic Cooperation and Development nations.^2^ U.S. life expectancy peaked in 2014 and has been decreasing for three consecutive years,^3^,^4^ a trend not seen since the influenza pandemic a century ago.^5^ Put simply, U.S. health is in decline.

This trend is uniquely American; lifespans in most high-income countries continue to climb. Other high-income countries have better birth outcomes, a lower prevalence of teen pregnancies, and lower mortality rates for cardiovascular disease, diabetes, and many other conditions. The U.S. health disadvantage is not for lack of spending on health care; for many years, the U.S. has vastly outspent other countries on health care.^6^ The explanation for why countries that spend less on health care have better health outcomes is obvious: health is about more than health care.

Access to health care, albeit essential, accounts for only 10%–20% of the variation in premature mortality.^7^ Health care is necessary but not sufficient to improve health, because the strongest influences on health are outside the clinic. Health behaviors (e.g., smoking, physical inactivity) are major factors, but behaviors are shaped by living conditions, not just personal choice. People can make only those choices available to them. It is difficult to eat fresh produce if it is not sold at local stores, to walk to work without a pedestrian route, or to have a colonoscopy without access to a gastroenterologist.

Upstream factors determine downstream conditions for good health. Chief among these is socioeconomic status (e.g., education, income). In a knowledge economy, education opens doors to economic opportunity and gives people the resources to pay medical bills, purchase nutritious foods, and live in healthy neighborhoods. Place matters to health because of how the physical and social environment affect us. The physical environment can harm health if the air and water are polluted, or if the built environment lacks green space, access to healthy foods, and affordable, quality housing and transportation. The social environment can also harm health, as when children are exposed to adverse childhood events^8^ or when people experience chronic stress, trauma, violence, social isolation, segregation, racism, or other forms of discrimination.

Low-income families and people of color have disproportionate exposure to these adverse conditions. Their children have less access to a good education and diminished social mobility—the chance to climb the economic ladder and fare better than their parents.^9^ To find stable housing and meet expenses, they must often settle in communities that lack resources for good health and expose them to risks, from toxic highway and power plant emissions to violence and crime.

These challenging conditions did not arise by chance. Health inequities, across populations and geographies, are the products not only of macroeconomic trends but also specific policies. Health is shaped less by doctors and scientists than by decision-makers in government and the private sector who determine employment opportunities, wages, land use, zoning, taxes, and education spending. Historic actions taken decades ago helped create today’s health inequities, and today’s policymakers have the power to close (or widen) the gaps. Policy decisions can harm health and weaken social mobility, as when they stifle local economies and claim jobs, or they can bring progress, as when investments in education stimulate economic opportunity. Policies catering to special interests have long afflicted rural America, leaving communities to struggle with stagnant economies, unemployment, persistent poverty, depopulation, and social isolation.

Nowhere is this better understood than in Appalachia, where the connections between history, the social determinants of health (e.g., poverty), and health inequities are conspicuous. The same issues intersect—sometimes more subtly—in pockets across the country, but nowhere has concentrated deprivation involved so large a region. Given the size of the region’s population, what affects Appalachia can shift national health trends.

This certainly applies to the current decline in U.S. life expectancy, the product of increasing mortality rates among young and middle-aged adults.^10^ Many of these “midlife” deaths have occurred in Appalachia. The increase in all-cause mortality among U.S. adults aged 25–64 years, which began in 2010, has been acute in West Virginia and surrounding states (Figure 1). Between 2010 and 2017, midlife all-cause mortality rates in West Virginia, Ohio, Kentucky, and Pennsylvania increased by 23.0%, 21.6%, 14.7%, and 14.3%, respectively (compared to less than 7.0% in southern Appalachian states).^11^ Between 2014 and 2016, life expectancy in these states (and Maryland) decreased by 0.5 years or more; only three other U.S. states (Arkansas, Maine, and New Hampshire) experienced so large a decrease.^12^

As part of a current study, we have estimated the number of excess deaths in the U.S. caused by the increase in midlife mortality during 2010–2017. The 13 Appalachian states (Alabama, Georgia, Kentucky, Maryland, Mississippi, New York, North Carolina, Ohio, Pennsylvania, South Carolina, Tennessee, Virginia, and West Virginia) represented one third (32.5%) of the U.S. population but accounted for half (49.6%) of these excess deaths. Roughly one third (31.3%) of excess deaths occurred in four Appalachian states—Ohio, Pennsylvania, Kentucky, and North Carolina. (Some New England states also experienced dramatic mortality increases but contributed less to excess deaths because of smaller populations.)

The leading cause of death responsible for increasing midlife mortality rates requires little imagination: accidental drug overdoses, fueled by the nation’s opioid epidemic, increased at alarming rates in Appalachia. Between 1999 and 2017, the rate of fatal drug overdoses in Pennsylvania, Kentucky, Ohio, and West Virginia increased by 660.4%, 929.1%, 1486.4%, and 3147.8%, respectively.

Midlife mortality rates also increased for other causes, including alcoholic liver disease and suicides, leading some to attribute the trend to “deaths of despair.” However, our research shows that midlife mortality rates in the U.S. are also increasing for a variety of organ diseases involving the circulatory, digestive, endocrine, neurologic, and many other body systems. Table 1 provides examples from Appalachian states.

No single factor—be it opioids, obesity, or firearms—could explain so pervasive a trend, causing drug addiction, suicides, a vast array of organ diseases, injuries, and even deaths from pregnancy and childbirth. The pervasiveness suggests the existence of upstream, systemic causes that could express themselves over time across diverse health outcomes.

More research is needed to pinpoint those root causes, but there are potential clues. For example, U.S. life expectancy began to lose pace with other countries in the 1980s, a time when income inequality began to widen in the U.S. Could income inequality—which has now reached epic proportions, exceeding that of other countries^13^—help explain the growing U.S. health disadvantage? Another potential clue lies in state life expectancy values, which began to diverge in the 1990s, when policies of devolution—shifting greater resources (e.g., block grants) and authorities to the states—began to take effect.^14^ Could the growing health gap between states reflect different choices by state governments, from public health legislation to pre-emption laws, Medicaid expansion, and investments in education and social services?

Moving from speculation to hard evidence about root causes requires research, and that research must be interdisciplinary. Understanding how the past produced current health inequities requires not only knowledge of public health and epidemiology but also demography, sociology, political science, history, economics, and other disciplines. Such interdisciplinary research is a priority: Lives are at stake. Knowing if flawed policies are culpable for the current health crisis is urgent. Unfortunately, investments in such research are paltry compared to spending on molecular biology or medical technology.

The price of neglecting this issue, in Appalachia and nationwide, is profound. The deaths of so many people in the prime of their lives will have broad ripple effects on the wellbeing of families and the social fabric of communities. The population at risk—adults aged 25–64 years—are working age, and thus the productivity and competitiveness of U.S. businesses and the economy are at risk. The resulting healthcare costs will burden not only government payers (e.g., Medicaid, Medicare) but also employers. Most of all, the trend threatens today’s children, whose parents are more likely to die prematurely and whose own life expectancy is jeopardized if corrective action is not taken.

With all this at stake, policymakers should not wait for more research—or for the health crisis to worsen—before taking action. We know enough about the social determinants of health to recognize the answers: promote education, create jobs that pay a living wage, invest in communities, and expand access to health care, affordable housing, and transportation. Doing this in Appalachia requires efforts to build infrastructure and attract new industries and jobs for a workforce sidelined by the collapse of the mining and manufacturing sectors. Policymakers who forego these efforts, whether for ideological reasons or to protect the profits of corporations and wealthy constituents, will save no lives. The U.S. health disadvantage will persist or worsen if the living conditions of the American populace do not improve.

## Figures and Tables

**Figure 1:**
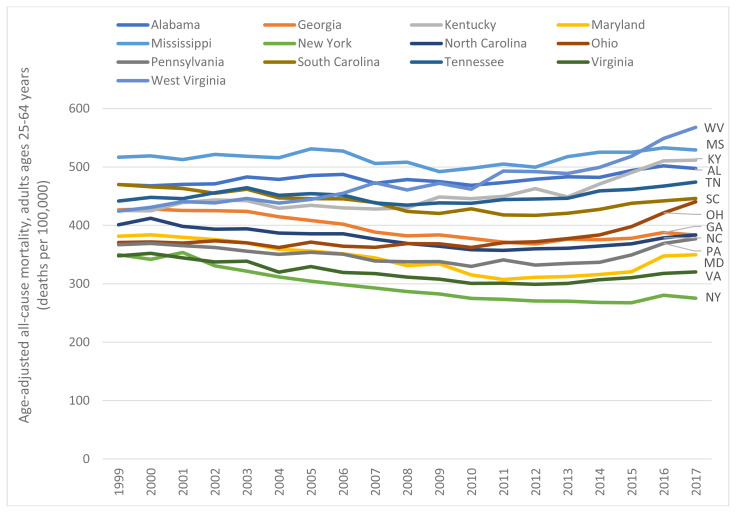
All-cause mortality among U.S. adults aged 25–64 years

**Table 1:** Relative increases in age-adjusted mortality rates between 1999 and 2017 by cause of death, adults ages 25–64 years, Appalachian states (N=13)

	Causes of death (ICD-10 codes)							
States	All-cause mortality	Accidental drug overdoses (X40–X44)	Alcoholic liver disease (K70)	Suicides (X60–84)	Hypertensive diseases (I10–I15)	Liver cancer (C22)	Obesity (E65–68)	Chronic lower respiratory disease (J40–J47)
AL	5.9% (469.7, 497.4)	577.2% (4.1, 27.9)	50.0% (4.5, 6.8)	44.5% (15.7, 22.7)	58.9% (8.7, 13.8)	33.5% (3.8, 5.1)	167.5% (1.2, 3.1)	37.0% (13.9, 19.0)
GA	−10.2% (426.9, 383.2)	436.8% (4.2, 22.6)	27.6% (4.9, 6.2)	32.9% (13.4, 17.9)	116.2% (8.6, 18.6)	77.6% (2.5, 4.4)	122.2% (1.4, 3.0)	−3.0% (13.4, 13.0)
KY	20.3% (425.5, 511.7)	929.1% (6.0, 61.9)	66.2% (5.5, 9.1)	50.9% (15.5, 23.4)	218.9% (5.0, 15.9)	86.5% (2.7, 5.1)	180.0% (1.4, 3.9)	35.7% (17.1, 23.2)
MD	−8.3% (381.5, 349.8)	1705.3% (1.0, 17.2)	−30.7% (5.3, 3.7)	27.3% (10.0, 12.7)	51.7% (10.9, 16.6)	88.6% (2.6, 4.9)	75.9% (1.7, 2.9)	−25.5% (8.5, 6.3)
MS	2.4% (516.9, 529.2)	410.4% (3.6, 18.1)	7.0% (5.5, 5.8)	42.8% (14.2, 20.2)	114.8% (12.0, 25.8)	36.7% (4.2, 5.8)	240.7% (1.5, 5.1)	46.8% (12.5, 18.4)
NY	−21.3% (349.8, 275.3)	289.2% (7.6, 29.5)	4.9% (5.4, 5.6)	39.3% (8.1, 11.2)	31.3% (8.2, 10.7)	20.5% (3.0, 3.7)	133.6% (1.1, 2.5)	−25.1% (9.1, 6.8)
NC	−4.4% (401.2, 383.7)	690.3% (4.7, 36.8)	14.9% (7.0, 8.0)	31.9% (14.4, 19.0)	62.8% (6.2, 10.2)	126.3% (2.1, 4.7)	154.9% (1.3, 3.4)	7.8% (12.3, 13.2)
OH	18.8% (370.8, 440.4)	1486.4% (4.9, 76.9)	84.2% (4.6, 8.5)	66.1% (11.9, 19.8)	109.3% (5.9, 12.4)	85.3% (2.4, 4.4)	150.0% (1.3, 3.2)	29.2% (11.9, 15.3)
PA	2.8% (366.8, 377.1)	660.4% (9.4, 71.1)	53.7% (3.3, 5.0)	45.5% (13.9, 20.3)	100.3% (4.0, 8.1)	80.1% (2.5, 4.5)	91.7% (1.2, 2.3)	−11.1% (10.1, 8.9)
SC	−5.1% (470.0, 446.0)	629.1% (4.4, 32.3)	6.5% (9.6, 10.2)	68.0% (13.6, 22.8)	23.8% (8.4, 10.4)	68.3% (3.3, 5.5)	60.8% (1.3, 2.0)	13.4% (13.8, 15.7)
TN	7.3% (441.8, 474.2)	622.3% (5.7, 41.5)	50.1% (6.2, 9.4)	32.7% (17.4, 23.0)	73.5% (8.6, 14.9)	135.8% (2.6, 6.1)	289.7% (1.1, 4.2)	33.3% (15.0, 20.0)
VA	−7.9% (347.9, 320.4)	391.6% (5.6, 27.3)	79.8% (3.3, 5.9)	18.9% (14.8, 17.6)	62.2% (5.1, 8.3)	34.6% (3.0, 4.0)	83.5% (1.2, 2.2)	−17.1% (10.2, 8.4)
WV	33.8% (424.4, 567.9)	3147.8% (3.0, 97.8)	62.4% (5.8, 9.5)	102.0% (15.3, 30.8)	436.8% (3.0, 16.1)	110.1% (2.4, 5.0)	NA (UR, 7.4)	76.8% (13.8, 24.3)

Source: Centers for Disease Control and Prevention, National Center for Health Statistics. Underlying Cause of Death 1999–2017 on CDC WONDER Online Database. Accessed at http://wonder.cdc.gov/ucd-icd10.html. *NA* = not applicable, *UR* = unreliable (deaths counts too low for stable estimates).
